# Electrochemical Evaluation of Tyrosinase Enzymatic Activity in Deep Eutectic Solvent and Aqueous Deep Eutectic Solvent

**DOI:** 10.3390/s23083915

**Published:** 2023-04-12

**Authors:** Rossella Svigelj, Fabiola Zanette, Rosanna Toniolo

**Affiliations:** Department of Agrifood, Environmental and Animal Science, University of Udine, Via Cotonificio 108, 33100 Udine, Italy; zanette.fabiola@spes.uniud.it

**Keywords:** deep eutectic solvents, enzyme activity, phenol, electrochemistry, biosensor

## Abstract

The use of green, inexpensive, and biodegradable deep eutectic solvents as nonaqueous solvents and electrolytes could be a useful way to potentially improve the enzyme biosensor performance as well as a profitable strategy to extend their use in the gas phase. However, enzyme activity in these media, although fundamental for their implementation in electrochemical analysis, is still almost unexplored. In this study, an electrochemical approach was employed to monitor tyrosinase enzyme activity in a deep eutectic solvent. This study was performed in a DES consisting of choline chloride (ChCl) as a hydrogen bond acceptor (HBA) and glycerol as a hydrogen bond donor (HBD), while phenol was chosen as the prototype analyte. The tyrosinase enzyme was immobilized on a gold-nanoparticle-modified screen-printed carbon electrode, and its activity was monitored following the reduction current of orthoquinone produced by the tyrosinase biocatalysis of phenol. This work represents a first step toward the realization of green electrochemical biosensors capable of operating in both nonaqueous and gaseous media for the chemical analysis of phenols.

## 1. Introduction

The use of alternative green solvents to conduct enzymatic reactions in partially or totally nonaqueous environments is of growing interest, especially when dealing with hydrophobic molecules or unstable enzymes [1]. In this context, many efforts have been made to develop and use environmentally friendly solvents known as Deep Eutectic Solvent (DESs). In 2004, Abbott introduced this new class of solvent which has attracted attention due to some interesting characteristics, such as low volatility and low toxicity [2]. DESs share similar properties with ionic liquids (ILs), a class of organic salts with a low melting point, usually obtained by combining an organic cation (commonly imidazolium-based cations) with a large variety of anions (i.e., Cl^−^, BF_4_^−^, PF_6_^−^, and NTf_2_) [3]. However, DESs have arisen as a cheaper and greener alternative [4,5].

DESs derive from the mixing, in appropriate stoichiometric ratios, of two components: a hydrogen bond acceptor (HBA) and a hydrogen bond donor (HBD) [6]. The final mixture has a melting point lower than the starting components.

In the analytical field, DESs have found use as extracting solvents in chromatographic and electrophoretic separations, and recently, they have also been used in the construction of electrochemical sensors [7,8,9]. In the electroanalytical field, they have been used simultaneously as preconcentrating electrolytes and solvents in the development of gas sensors [10,11] and biosensors [12].

For biosensors, enzymes capable of catalyzing certain reactions are often used, and although water represents the best green solvent, its high polarity hinders its use in many biocatalytic reactions, since many substrates are not water soluble [13]. Moreover, both in the sensors and biosensors field, it is common to use low volumes of water which lead to a rapid evaporation of the solvent, thus generating problems in the measurements, especially when working in the gas phase.

Several enzymes have been successfully used in DES or in “water-in-DES” systems to catalyze reactions; in fact, recent studies have shown that some enzymes, that are not stable in aqueous solutions of the single constituents of a DES, are conversely stable in the corresponding DESs [14]. This different behavior is probably attributable to the network of hydrogen bonds formed in the DES, which reduces the chemical reactivity of the starting constituents [15]. Furthermore, DESs involve a modification of the tertiary structure of enzymes, increasing the alpha-helix secondary structure [16]. This action gives greater stability to the enzyme structure, which results in an increased enzymatic activity.

Several studies related to enzymatic processes in DES have been reported in the literature. For example, a DES consisting of choline acetate and glycerol has been successfully employed for lipase-catalyzed transesterification reactions [17,18].

In the biocatalytic field, DESs have been mainly used as pure solvents, without the addition of water or other co-solvents [19]. Molecular dynamics simulations have been conducted for some water–DESs systems highlighting that water in small amounts is adsorbed in the structure of the DESs, and the hydrogen bond interactions of the liquid do not change up until a 50% water fraction [20].

In this study, an electrochemical approach was employed to monitor tyrosinase enzyme activity in a DES consisting of choline chloride (ChCl) as the hydrogen bond acceptor (HBA) and glycerol (Gly) as the hydrogen bond donor (HBD) (ChCl-Gly, 1:2). Phenol was chosen as the analyte as it is the basic molecule of phenolic compounds, which are a group of naturally occurring compounds found in a wide range of foods, such as fruits, vegetables, grains, legumes, nuts, and spices. These compounds are indicators of the quality and freshness of food products, and their analysis can be used to determine the ripeness, storage, and processing conditions of food.

Therefore, to evaluate the enzymatic activity in the DES ChCl-Gly, tyrosinase enzyme was immobilized on a gold-nanoparticle-modified Screen-Printed Carbon Electrode (SPCE), and its activity was monitored following the reduction current of orthoquinone produced by tyrosinase biocatalysis of phenol. This work represents a first step toward the realization of green electrochemical biosensors capable of operating in both nonaqueous and gaseous media.

## 2. Materials and Methods

### 2.1. Materials

Choline chloride, glycerol, potassium phosphate monobasic, sodium phosphate dibasic, tyrosinase enzyme, potassium ferrocyanide, gluteraldehyde, and phenol were purchased from Sigma-Aldrich (Milan, Italy). Tetrachloroauric acid (HAuCl_4_) was purchased from Johnson Matthey Chemicals (Cambridge, UK). For the preparation of all the solutions, ultrapure water (R > 18 MΩ) was used, obtained by means of an Elga Purelab flex 4 system (Veolia Water Technologies, Zoppola, Italy). The same water was also used for the cleaning and rinsing operations. The electrochemical measurements were conducted using screen-printed carbon electrodes (SPCEs) manufactured by Dropsens (Metrohm Italiana S.r.l., Varese, Italy).

All voltametric and chronoamperometric measurements were performed using an Autolab PGSTAT204 potentiostat (Metrohm Italiana S.r.l., Varese, Italy) managed by Nova version 2.2 software and connected to the SPCE by means of a Dropsens model CAC connector cable (Metrohm Italiana S.r.l., Varese, Italy).

### 2.2. DES Synthesis

A DES composed of choline chloride and glycerol (ChCl-Gly), in a stochiometric ratio of 1:2, was prepared by mixing the HBA and the HBD in molar ratios following a protocol reported in the literature [15]. Briefly, the mixture was placed under stirring and heated at a temperature of 80 °C for two hours.

### 2.3. Modification of the SPCE with Tyrosinase and Activity Measurements

**Figure 1:**
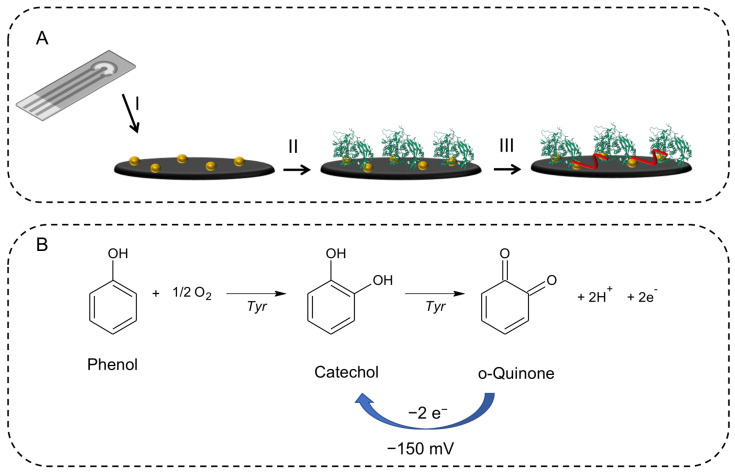
(**A**) Schematic representation of the working electrode modifications: (I) electrogeneration of the AuNPs using a solution of tetrachloroauric acid and subsequent reduction of Au(III) by applying a potential equal to +0.18 V to the working electrode for 50 s, (II) immobilization of tyrosinase enzyme (100 IU/μL), (III) cross-linking reaction using a 25% gluteraldehyde solution. (**B**) The oxidation process of phenol to ortho-quinone catalyzed by tyrosinase.

The electrochemical test used to evaluate the enzymatic activity of tyrosinase was carried out using a modified SPCE by adapting a protocol described elsewhere [21], and it is schematically represented in Figure 1A. Firstly, the electrochemical generation of gold nanoparticles (AuNPs) on the surface of the SPCE was carried out, by deposition of 60 μL of a solution of tetrachloroauric acid and subsequent reduction of Au (III) by applying a potential equal to +0.18 V against the Ag pseudo reference to the working electrode for 50 s [22]. Then, 5 μL of a 100 IU/μL solution of tyrosinase was drop-casted on the working electrode and left to evaporate. Lastly, 5 μL of a 25% gluteraldehyde solution was deposited on the working electrode for 30 min to crosslink the enzyme on the electrode surface. After a washing step, the sensor was ready to use.

Tyrosinase-based biosensors utilize the activity of tyrosinase, which catalyzes the oxidation of phenols, to detect the presence of certain compounds. When phenol is introduced into the biosensor, it reacts with the immobilized tyrosinase to produce in the presence of molecular oxygen, as shown in Figure 1B, firstly catechol and then a two-electron oxidation that converts catechol into orto-quinone. The quinone may be reduced back to catechol by applying an adequate potential. In fact, we have electrochemically reduced the orthoquinone obtaining a signal proportional to the phenol concentration. By constructing various calibration lines in mixtures of DES-PBS buffer, we were able to evaluate the enzyme activity based on the slope of the calibrations obtained.

### 2.4. Electrochemical Measurements

To define the electrochemical window of the DES and DES-PBS solutions, a voltametric analysis was conducted by depositing 60 μL of each medium investigated on the SPCE. Then, 1 mM solutions of phenol in DES-PBS were used to establish its voltametric behavior.

The enzymatic activity was evaluated using a chronoamperometric test conducted on modified SPCEs by applying to the working electrode a potential of −150 mV against a Ag pseudo reference for 60 s to electrochemically reduce all the orthoquinone generated by the enzyme.

## 3. Results and Discussion

### 3.1. Electrochemical Characterization

Firstly, an electrochemical investigation of the useful potential window in ChCl-Gly was carried out on the bare SPCE. The potential window useful for electrochemical measurement in 50% *v*/*v* solutions of DES-PBS buffer is between about −0.4 V and +1.0 V. This compared with that recorded in phosphate buffer, appears to be similar in both anodic and cathodic potentials.

**Figure 2:**
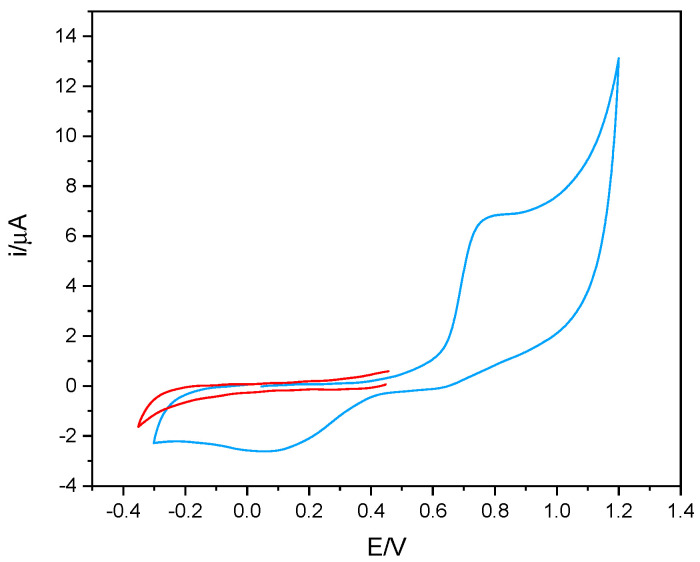
The voltametric behavior of 1 mM phenol in 50% *v*/*v* DES-PBS solution (blue line) and cathodic scan of 1 mM phenol in 50% *v*/*v* DES-PBS buffer solution (red line). Potential scan rate of 50 mV/s.

Considering the measurement strategy of the enzymatic activity of tyrosinase, based on the electrochemical reduction of the quinone product, the voltametric behavior of phenol was also studied. By way of example, Figure 2 shows the voltametric behavior of phenol recorded in 50% *v*/*v* solutions of DES-PBS buffer. As can be seen, phenol presents an oxidation process around 0.8 V and the relative reduction peak to the potential of about 0.1 V. Furthermore, it can be observed that, by appropriately choosing the initial potential of the cathodic scan, no reduction process was observed (red line). This result indicates that to monitor the enzymatic activity of tyrosinase by amperometric measurement, all the corresponding potential values between 0 and −0.2 V can be used, since in the absence of the enzyme, no electrochemical process was present at these potentials.

### 3.2. Enzymatic Activity Evaluation

The enzymatic activity was evaluated using a chronoamperometric test conducted on modified SPCEs. For this purpose, phenol solutions of known concentration (0, 5, 10, 15, and 25 μM) in 0.1 M phosphate buffer at pH 6.5 and in DES-PBS solutions with increasing DES content were used. Then, 60 μL of these solutions with an increasing concentration of phenol was deposited on the modified SPCE, and after 90 s of incubation, we proceeded with the chronoamperometric measurement by applying to the working electrode a potential of −150 mV vs. Ag pseudo reference for 60 s. At this potential, the reduction current of the orthoquinone produced starting from the phenol was recorded thanks to the activity carried out by the tyrosinase immobilized on the surface of the electrode, as shown in Figure 1B.

**Figure 3:**
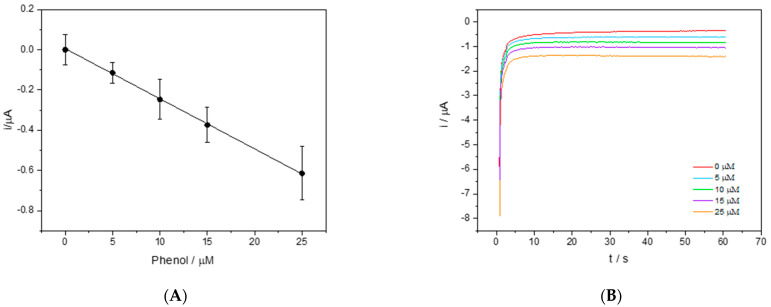
(**A**) The calibration line of phenol in 50% *v*/*v* DES-PBS solutions. The chronoamperometric measurement was obtained by applying to the working electrode a potential of −150 mV vs. the Ag pseudo reference for 60 s. (**B**) The chronoamperometric curves at different concentrations of phenol (0, 5, 10, 15, and 25 μM).

Figure 3A shows the relative calibration line in 50% DES-PBS obtained after 90 s of contact between the enzyme present on the electrode surface and the solutions with a known concentration of phenol. Figure 3B shows the corresponding chronoamperometric curves.

**Figure 4:**
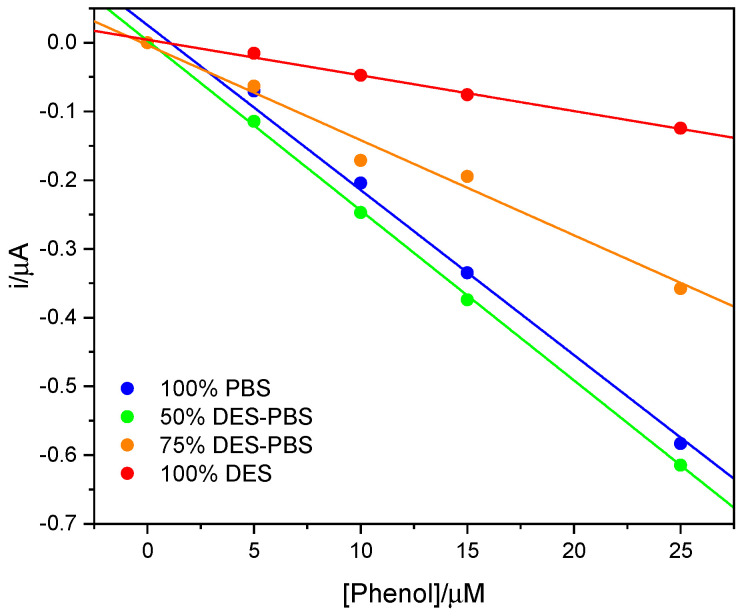
The calibration lines of the phenol in 100% PBS (blue line), 50% *v*/*v* DES-PBS (green line), 75% *v*/*v* DES-PBS (orange line), and 100% DES (red line).

The tyrosinase activity was then compared to that obtained in buffer and at higher percentages of DES, as can be seen in Figure 4. The calibration lines were obtained from the measurements made in triplicate; for clarity, the error bars are not shown in the figure, but the standard deviation fell between ±0.01 and ±0.13 μA. It can be observed that at 50% DES, the enzymatic activity was maintained and was comparable to that obtained in buffer, while at higher quantities of DES a decrease in activity was noted; however, the enzyme maintained its ability to catalyze the reaction.

In Table 1, the slopes of the calibration lines, the coefficients of determination, and the limits of detection are reported. Figure 4 shows that the enzyme worked in a comparable way at 50% of DES compared to the buffer alone, while the response gradually decreased with an increase in the amount of DES, remaining active even at 100% DES. This behavior could be attributable to the higher viscosity of DES. At the same time, the evaluation of apparent K_m_, obtained applying the Lineweaver–Burk plot, led to comparable values for 100% PBS and 100% DES. Similar K_m_ values may indicate that the enzyme activity remained the same in both solvents, while the lower sensitivity in the DES could be attributable to the higher viscosity of the DES and therefore to a more difficult diffusion towards the electrode surface during the electrochemical measurement.

The fact that the enzyme is able to work in the DES could be due to the unique chemical and physical properties of this class of solvents. The chemical nature of the DESs can help stabilize enzymes and prevent the loss of enzyme activity due to changes in the environmental conditions such as the temperature, pH, or ionic strength [23]. DESs can also help to increase the solubility of the substrate molecules, allowing for improved substrate binding and catalytic activity [13]. Furthermore, DESs can modulate the hydrophobic and electrostatic interactions between the enzymes and the substrates, leading to improved catalytic efficiency.

Overall, the combination of the unique properties of DESs, such as their low melting point, biocompatibility, ability to dissolve a wide range of substances, and ability to stabilize enzymes, make them promising solvents for enzyme-catalyzed reactions.

The results obtained are very important for the chemical analysis of phenols; in fact, they may open the possibility of implementing gas sensors for the analysis of these chemical species in different contexts.

## 4. Conclusions

In recent years, researchers have explored the use of DESs as media for enzymes in biocatalysis, due to the ability of DESs to dissolve both water-soluble and water-insoluble substrates. This allows for greater accessibility of the enzymes to their substrates, which can increase their activity and efficiency. Additionally, DESs can also serve as a source of cofactors, which are essential for the activity of many enzymes [24].

The results obtained in this exploratory study may represent a valid starting point for the realization of electrochemical enzyme biosensors capable of monitoring phenolic compounds. As regards the application in liquid samples, the good functionality of tyrosinase in 50% DES-PBS buffer mixtures, can be useful in the development of electrochemical biosensors considering the extraction and preconcentrating capabilities of these means. Moreover, it is important to highlight that the capacity of tyrosinase enzyme to retain its activity also in 100% DES, could be a key result for the successful development of enzymatic biosensors operating in gas phase thanks to DESs’ low volatility and ability to act as electrolytes and solvents.

In conclusion, combining the features of DESs and enzymes has great potential not only in biosensor technology but also in bioconversion, bioremediation, and the synthesis of bio-based chemicals.

## Figures and Tables

**Table 1:** The slopes of the calibration lines, the coefficients of determination, and the limits of detection for each calibration line.

	Slope	R^2^	LOD (μM)
100% PBS	−0.024	0.99	0.35
50% DES-PBS	−0.025	0.99	0.47
75% DES-PBS	−0.013	0.95	5.66
100% DES	−0.005	0.98	6.46

## Data Availability

The data presented in this study are available on request from the corresponding author.

## References

[1] Gotor-Fernández V., Paul C.E. (2019). Deep Eutectic Solvents for Redox Biocatalysis. J. Biotechnol..

[2] Abbott A.P., Boothby D., Capper G., Davies D.L., Rasheed R.K. (2004). Deep Eutectic Solvents Formed between Choline Chloride and Carboxylic Acids: Versatile Alternatives to Ionic Liquids. J. Am. Chem. Soc..

[3] Galiński M., Lewandowski A., Stępniak I. (2006). Ionic Liquids as Electrolytes. Electrochim. Acta.

[4] Deetlefs M., Seddon K.R. (2010). Assessing the Greenness of Some Typical Laboratory Ionic Liquid Preparations. Green Chem..

[5] Vanda H., Dai Y., Wilson E.G., Verpoorte R., Choi Y.H. (2018). Green Solvents from Ionic Liquids and Deep Eutectic Solvents to Natural Deep Eutectic Solvents. Comptes Rendus Chim..

[6] Abbott A.P., Capper G., Gray S. (2006). Design of Improved Deep Eutectic Solvents Using Hole Theory. Chem. Eur. J. Chem. Phys..

[7] Cai T. (2019). Application of Deep Eutectic Solvents in Chromatography: A Review. Trends Anal. Chem..

[8] Abbott A.P. (2022). Deep Eutectic Solvents and Their Application in Electrochemistry. Curr. Opin. Green Sustain. Chem..

[9] Svigelj R., Dossi N., Grazioli C., Toniolo R. (2021). Deep Eutectic Solvents (DESs) and Their Application in Biosensor Development. Sensors.

[10] Buzzeo M.C., Hardacre C., Compton R.G. (2004). Use of Room Temperature Ionic Liquids in Gas Sensor Design. Anal. Chem..

[11] Toniolo R., Dossi N., Giannilivigni E., Fattori A., Svigelj R., Bontempelli G., Giacomino A., Daniele S. (2020). Modified Screen Printed Electrode Suitable for Electrochemical Measurements in Gas Phase. Anal. Chem..

[12] Svigelj R., Dossi N., Grazioli C., Toniolo R. (2021). Paper-Based Aptamer-Antibody Biosensor for Gluten Detection in a Deep Eutectic Solvent (DES). Anal. Bioanal. Chem..

[13] Schweiger A.K., Ríos-Lombardía N., Winkler C.K., Schmidt S., Morís F., Kroutil W., González-Sabín J., Kourist R. (2019). Using Deep Eutectic Solvents to Overcome Limited Substrate Solubility in the Enzymatic Decarboxylation of Bio-Based Phenolic Acids. ACS Sustain. Chem. Eng..

[14] Xu P., Zheng G.-W., Zong M.-H., Li N., Lou W.-Y. (2017). Recent Progress on Deep Eutectic Solvents in Biocatalysis. Bioresour Bioprocess.

[15] Zhang Q., De Oliveira Vigier K., Royer S., Jerome F. (2012). Deep Eutectic Solvents: Syntheses, Properties and Applications. Chem. Soc. Rev..

[16] Wu B.-P., Wen Q., Xu H., Yang Z. (2014). Insights into the Impact of Deep Eutectic Solvents on Horseradish Peroxidase: Activity, Stability and Structure. J. Mol. Catal. B Enzym..

[17] Gorke J.T., Srienc F., Kazlauskas R.J. (2008). Hydrolase-Catalyzed Biotransformations in Deep Eutectic Solvents. Chem. Commun..

[18] Zhao K.-H., Cai Y.-Z., Lin X.-S., Xiong J., Halling P.J., Yang Z. (2016). Enzymatic Synthesis of Glucose-Based Fatty Acid Esters in Bisolvent Systems Containing Ionic Liquids or Deep Eutectic Solvents. Molecules.

[19] Arnodo D., Maffeis E., Marra F., Nejrotti S., Prandi C. (2023). Combination of Enzymes and Deep Eutectic Solvents as Powerful Toolbox for Organic Synthesis. Molecules.

[20] Gabriele F., Chiarini M., Germani R., Tiecco M., Spreti N. (2019). Effect of Water Addition on Choline Chloride/Glycol Deep Eutectic Solvents: Characterization of Their Structural and Physicochemical Properties. J. Mol. Liq..

[21] Njagi J., Warner J., Andreescu S. (2007). A Bioanalytical Chemistry Experiment for Undergraduate Students: Biosensors Based on Metal Nanoparticles. J. Chem. Educ..

[22] Svigelj R., Zuliani I., Grazioli C., Dossi N., Toniolo R. (2022). An Effective Label-Free Electrochemical Aptasensor Based on Gold Nanoparticles for Gluten Detection. Nanomaterials.

[23] Huang Z.-L., Wu B.-P., Wen Q., Yang T.-X., Yang Z. (2014). Deep Eutectic Solvents Can Be Viable Enzyme Activators and Stabilizers: DESs Can Be Enzyme Activators and Stabilizers. J. Chem. Technol. Biotechnol..

[24] Chanquia S.N., Huang L., García Liñares G., Domínguez de María P., Kara S. (2020). Deep Eutectic Solvents as Smart Cosubstrate in Alcohol Dehydrogenase-Catalyzed Reductions. Catalysts.

